# Immunohistochemical Expression of Ornithine Decarboxylase, Diamine Oxidase, Putrescine, and Spermine in Normal Canine Enterocolic Mucosa, in Chronic Colitis, and in Colorectal Cancer

**DOI:** 10.1155/2015/172756

**Published:** 2015-10-15

**Authors:** Giacomo Rossi, Matteo Cerquetella, Graziano Pengo, Subeide Mari, Emilia Balint, Gabrio Bassotti, Nicolae Manolescu

**Affiliations:** ^1^School of Biosciences and Veterinary Medicine, University of Camerino, Via Circonvallazione 93/95, 62024 Matelica, Italy; ^2^S. Antonio Clinic, 26020 Madignano, Italy; ^3^Faculty of Veterinary Medicine, 050097 Bucharest, Romania; ^4^Gastroenterology and Hepatology Section, Department of Medicine, Santa Maria della Misericordia Hospital, Piazzale Menghini 1, San Sisto, 06153 Perugia, Italy

## Abstract

We compared the immunohistochemical expression of putrescine (PUT), spermine (SPM), ornithine decarboxylase (ODC), and diamine oxidase (DAO) in bioptic samples of canine colonic mucosa with chronic inflammation (i.e., granulomatous colitis and lymphoplasmacytic colitis) or neoplasia. Single and total polyamines levels were significantly higher in neoplastic tissue than in normal samples. Samples with different degrees of inflammation showed a general decrease expression of ODC if compared to controls; SPM was practically not expressed in control samples and very low in samples with chronic-granulomatous inflammation. In carcinomatous samples, the ODC activity was higher with respect to controls and samples with inflammation. This is the first description of polyamines expression in dog colonic mucosa in normal and in different pathological conditions, suggesting that the balance between polyamine degradation and biosynthesis is evidently disengaged during neoplasia.

## 1. Introduction

The polyamines (PAs) spermine (SPM) and putrescine (PUT) are intimately involved in regulation of DNA, RNA, and protein synthesis; therefore, they are essential for proliferation of both normal and neoplastic cells. Dysregulation of cellular polyamines is associated with various pathological conditions, including inflammation, and cancer; for this latter association, polyamine pathways have been explored as targets for cancer chemotherapy and chemoprevention [1, 2]. Fujiwara et al. [3] reported the first immunohistochemical demonstration of PAs distribution in different portions of healthy gut mucosa of rats and mice using specific monoclonal antibodies and observed that PAs are well expressed in gastrointestinal tract according to the rapid turnover of gastrointestinal epithelium. The intracellular amount of PAs is the result of the biosynthetic activity of the key-enzyme ornithine decarboxylase (ODC) and of the uptaking from extracellular environment. In human beings several studies reported an increased expression of ODC in neoplastic colorectal tissue versus normal-appearing mucosa [4, 5].

The enzyme diamine oxidase (DAO) inactivates histamine and other biogenic amines, such as PAs by a reaction of oxidative deamination. DAO is mainly expressed in the intestine, located almost exclusively in the villus tip enterocytes of mammals [6]. Decreased levels of DAO were found in various bowel diseases both in dogs and in humans [6, 7]. Although polyamines are critical for optimal cell growth, excessive accumulation may interfere directly with normal cell function, and they have been implicated recently in the control of the apoptotic response, inflammation, and cancerogenesis [8, 9].

The present study was undertaken with an objective to evaluate, by means of immunohistochemistry, the localization, the pattern, and the levels of expression of polyamines SPM, PUT, ODC, and DAO in dog. For this purpose, we have compared results obtained from biopsy samples taken from clinically healthy dogs, and dogs with clinical and histological signs of large bowel pathology (ranging from chronic inflammation to neoplasia), to evaluate the significance of polyamines within the proliferative process and their possible role as prognostic markers and target of antiproliferative drugs.

## 2. Materials and Methods

### 2.1. Animals

Forty dogs participated in the study, after histological evaluation and inclusion in four different groups (control (group 1), granulomatous colitis (group 2), lymphoplasmacytic colitis (group 3), and colonic adenocarcinoma (group 4) consisting of 10 dogs each) (Table 1).

Dogs with histological diagnosis of intestinal lymphoma were excluded from the study, because the completely flat mucosa, showing crypt atrophy or hypertrophy and reduced epithelial cell number and height, with important alterations of epithelium showing colonocytes loss related to lymphocytes and neutrophils infiltration, could reduce the degree of polyamines expression. All enrolled dogs, excluding control animals, showed a long-time diagnosis of IBD according to published criteria [10] and were evaluated at the Veterinary Teaching Hospital, Camerino University, for chronic gastroenteritis. Inclusion criteria included recurrence of clinical signs and absence of any immunomodulating drug therapy (e.g., corticosteroids, metronidazole, and sulfasalazine) within a month before referral. Furthermore, for dogs of group 3 the diagnostic plan was integrated as expected while diagnosing inflammatory bowel diseases [11]. Diagnostic criteria for IBD included persistent (>3 weeks) gastrointestinal signs, failed responses to dietary (hydrolysate or commercial intact protein elimination diet) or symptomatic therapies (anthelmintics, antibiotics, anticholinergics, and gastrointestinal protectants) alone, a thorough diagnostic evaluation with failure to document other causes for gastroenteritis, and histopathologic evidence of intestinal inflammation. The minimum diagnostic evaluation in all dogs included a complete blood count, serum biochemistry, urinalysis, direct (wet mount) and indirect (flotation) examination of feces for endoparasites, and survey abdominal radiographs. In some instances, additional tests including contrast radiography, abdominal ultrasound (performed in 22 of the 30 pathologic dogs), and measurement of serum concentrations of trypsin-like immunoreactivity and/or folate and cobalamin were performed. Additional inclusion criteria were the absence of extra-alimentary tract inflammation based on results obtained from initial diagnostic testing. Dogs with hypoproteinemia or a suspicion of intestinal lymphangiectasia were excluded from the study. The clinical disease activity (CIBDAI score) was assessed at the enrollment. The CIBDAI is based on 6 criteria, each scored on a scale from 0 to 3: attitude/activity, appetite, vomiting, stool consistency, stool frequency, and weight loss. After summation, the total composite score is determined to be clinically insignificant (scores 0–3), mild (scores 4-5), moderate (scores 6–8), or severe (score 9 or greater) [12].

The control group was composed of dogs hospitalized in urgency and that died for different causes (mainly for severe trauma) but that were free of gastrointestinal signs for at least four months, not presenting gastrointestinal signs before death and in which necropsy did not reveal neoplastic conditions. Control dogs were considered “to be healthy,” based on clinical history, normal results on physical examination (excluding lesions due to trauma), serum biochemistry, urinalysis, fecal examinations, and* Dirofilaria* antigen assay performed immediately before or after death. All the owners of the IBD dogs gave informed written consent before enrollment.

#### 2.1.1. Tissue Sampling

After enrollment, multiple (10–15 specimens) mucosal biopsy specimens were procured endoscopically from the large intestine of all dogs (*n* = 30, 10 dogs per group of disease). Naturally deceased or humanely suppressed dogs (for the severity of the condition and independent of the study) were sampled directly, during the necropsy procedures, performed immediately after death. In dogs undergoing endoscopy, food administration was suspended around 36–48 hours before endoscopy, and dogs were prepared following standard protocols. Dogs of groups 2, 3, and 4 underwent a colonoscopy under general anesthesia (video-endoscope (Olympus EVIS GIF-100, Tokyo, Japan) (outer diameter, 9.5 mm; biopsy channel diameter, 2.8 mm)); a set of a minimum of 8 biopsies was taken from each enterocolic tract. In dogs of group 3, additional bioptic samples were taken in neoplastic areas. All dogs showed predominantly mixed signs of enterocolitis (i.e., GI signs associated with tenesmus, hematochezia, mucoid feces, and/or frequent defecation), and upper and lower endoscopic examinations were performed. Biopsy specimens were obtained directly from mucosal lesions of increased granularity, friability, or erosions as well as areas of normal-appearing mucosa. Tissues for histopathology were fixed in 4% buffered formaldehyde and then paraffin embedded and serial 4 *μ*m thick sections were prepared. Sections were cut, dewaxed, and stained with hematoxylin-eosin (H&E). Adjacent sections were subjected to immunohistochemical analysis (IHC) using a set of polyclonal antibodies. Histopathology was performed by a single pathologist, who was blinded regarding history, clinical signs, or endoscopic observations. A severity score was assigned for each dog, by using a standardized and previously described histologic grading system, based on the extent of architectural disruption and mucosal epithelial changes [12, 13], as has recently been proposed by the WSAVA for diagnosis of gastrointestinal inflammation [14].

### 2.2. Histopathology, Histochemistry, and Immunohistochemistry

Paraffin sections were rehydrated and neutralized for endogenous peroxidases with 3% hydrogen peroxide for 5 minutes followed by rinsing for 5 minutes in distilled water. For antigen retrieval, slides were incubated in EDTA buffer, pH 9.0, and processed in a microwave oven at 650 W for two cycles of 10 minutes each to unmask antigens. Slides were then allowed to cool at room temperature for at least 20 minutes before being processed for immunostaining by standard procedures. Tissue sections were incubated overnight in a moist chamber at 4°C with different primary antibodies (Abs): rabbit polyclonal Ab against ODC (*Bioss antibodies*, pAb rb-anti ODC antibody,* bs-1294R*; diluted 1 : 50), DAO (*Biorbyt*; pAb rb-anti DAO antibody,* orb192676*; diluted 1 : 100), PUT (*Thermo Fisher Scientific*; pAb rb-anti Pentane-1,5-diamine, #PA1-86537, diluted 1 : 20), and SPM (*Abcam*, ab7318, diluted 1 : 20) antigens. All primary antibodies were diluted in Tris-buffered solution (TBS) containing 0.1% crystalline bovine serum albumin (BSA). Tissue sections were incubated overnight in a moist chamber at 4°C with different primary antibodies, diluted (1 : 50). The antigens-antibodies complex was detected by ABC-peroxidase technique using 3-3′-diaminobenzidine-hydrochloride (Vector Laboratories, Inc., Burlingame, CA) as chromogen substrate to reveal the immunoreaction, with Meyer Haematoxylin as nuclear counterstain.

Nonspecific binding was blocked by incubation of slides for 10 minutes with a protein-blocking agent (protein-blocking agent, Dako, Carpinteria, CA, USA) before application of the primary antibody. Specific primary antibodies substituted with TBS or nonimmune sera were used as negative controls in immunohistochemical techniques.

The antibodies used were not validated for canine tissue, but the specificity of the reaction was assured by the fact that polyamines, consisting in polycationic molecules with multiple amino groups, and their cellular receptor are identical in both prokaryotic and eukaryotic cells [15]. Canine polyamines present the same structure of man polyamines and this reinforces the specificity of reaction. Finally, to test the specificity of antibodies used in our experiments, and the possibility of cross-reaction to each other, a western blot was performed as standardized by Kurien et al., 2011 [16]. In brief, different polyamines (Sigma) were loaded in equal amounts of samples onto 3–8% Tris-acetate gels and separated by electrophoresis (100 V for 1.5 h). The proteins were transferred to a Hybond-ECL Nitrocellulose membrane (GE Healthcare Bio-Sciences Corp., Piscataway, NJ, USA) and then immersed in a block solution with 5% dry milk in PBS for 1.5 h at room temperature. The different molecules were detected with the specific antibody employed also in IHC tests, used at a concentration of 1 : 5000 in 5% milk solution, and incubated overnight at 4°C. After washing in a Tris-buffered saline with 0.1% Tween (TBS-T) buffer and incubating for 45 min with a secondary antibody (horseradish peroxidase-conjugated goat anti-rabbit IgG; 1 : 5000), positively stained bands were detected by a chemiluminescent blot assay with the ECL Plus western blot reagent.

Histopathologic examination of all biopsies was performed by a single pathologist, who graded endoscopic specimens and assigned a lesion severity score for each dog by using a standardized and previously described histologic grading system, based on the extent of architectural disruption and mucosal epithelial changes [12, 13, 17, 18]. Briefly, the histologic examination of H&E-stained sections included the assessment of the number of inflammatory cells, using a visual analogue scale modified for canine specimens [19]. Number of inflammatory cells (mononuclear cells such as lymphocytes, plasma cells, macrophages, and neutrophils) was assessed at 400x magnification (high-power field (HPF)). Number of lymphocyte aggregates was assessed at 100x magnification. The number of inflammatory cells was recorded and results were reported as the mean for the entire specimen.

Histologic criteria for normal colonic mucosa included detection of none or only a few mononuclear cells scattered in the chorion per HPF, absence of lymphoid aggregates, and none or only a few scattered neutrophils across the intestinal epithelium.

In pathological samples, neutrophils were considered as absent (score 0) when there was none or only single sporadic cells per high-power field (HPF, using 400x magnification), mild (score 1) for a few cells (5 to 10 cells) per HPF, moderate (score 2) for several cells (11 to 50 cells) per HPF, or severe (score 3) (50 to 200 cells or more) per HPF. Number of mononuclear cells was considered to be normal when none or only few cells (<5 cells) were visible in each HPF among intestinal glands (score 0). Increasing in number of cells was considered mild for specimens with several cells per HPF (score 1 = 5 to 10 cells), moderate (score 2 = 11 to 30) for specimens with many cells for HPF, or severe (score 3 > 30 cells) for specimens with conspicuous and very conspicuous number of cells per HPF, respectively. Number of lymphocytic aggregates in a specimen was counted.

Finally colonic adenocarcinomas were described according to Head et al. [20, 21] on the basis of the current WHO's classification for tumours in domestic animals. The morphological types of neoplastic samples we examined were classifiable as tubular, well, middle, and poorly differentiated and mucinous, middle differentiated.

#### 2.2.1. Histochemistry

In the second step, a histochemical staining was performed on the same sections previously subjected to the histological and immunohistochemical assay. The histochemical staining was performed with only minor modifications according to Poletti et al. [22]. For Alcian blue staining, the sections were stained with Alcian blue solution (pH 2.5) for 30 min at room temperature, washed in running tap water for 10 min, rinsed in DI water, counterstained in Mayer's hematoxylin for 5 min, and washed in DI water. The acetic acid as mordant was not used because it has no effect on the final results. For PAS staining, sections were oxidized in 0,5% periodic acid solution for 5 min, rinsed in DI water, placed in Schiff reagent for 15 min, washed in tap water for 5 min, counterstained in Mayer's hematoxylin for 1 min, and washed in DI water. Finally, the slides were dehydrated, cleared, and mounted after differentiation with hydrochloric acid. To evaluate the localization and intensity of Alcian/PAS stain, the same method described also for immunohistochemical evaluation was used (see below).

#### 2.2.2. Immunohistochemistry

In immunohistochemical essays, the number of immunoreactive cells for each antibody observed in normal or pathological colonic samples was calculated using a light microscope (Carl Zeiss), a ×40 objective, a ×10 eyepiece, and a square eyepiece graticule (10 × 10 squares, with a total area of 62 500 *μ*m^2^). Ten appropriate sites were chosen for each colonic biopsy and arithmetic means were calculated for each colonic area. Results were expressed as IHC positive cells per 62 500 *μ*m^2^.Cells on the margins of the tissue sections were not considered for evaluation to avoid inflation of positive cell numbers.

To assess the intensity of polyamines expression (ODC, DAO, PUT, and SPM) in biopsies sampled of all groups and to compare data to the polyamines expression in healthy control dogs (group 1) stained tissue sections, ×250 photos were used. Photographs were evaluated by two blinded investigators who scored both the extent and intensity of staining in the lining and crypts epithelium of each picture on a scale of 0–3 (0 = absent and 3 = severe). The score of extent and intensity of staining were added and the mean of the total scores was calculated and used for analysis. The ×400 photographs were used to calculate the amount of immunostaining present in each section. One examiner opened each photograph in ImageJ (http://rsb.info.nih.gov/ij/) and a pixel intensity threshold was determined to include only those image pixels in immunopositive areas. For evaluation, colonic epithelium was divided into luminal, proximal, and distal gland/crypt regions. Finally, the scoring of colonic molecules expression was calculated as previously described.

### 2.3. Statistical Analysis

Differences between groups were assessed by nonparametric tests, using the Friedman test to evaluate variance and then the Wilcoxon test for paired samples. Values of *p* < 0.05 were chosen for rejection of the null hypothesis.

## 3. Results

### 3.1. Histopathology and Histochemistry

In chronic-granulomatous colitis (group 2), a general altered morphological status of the epithelial cells was observed, with flattened aspect of some areas of epithelium and epithelial cells loss, particularly in lining superficial epithelium; additionally an evident decrease in mucous goblet cells was found, comparing samples with normal dogs or dogs with LPC. Additionally, more pyknotic and karyorrhectic epithelial cells occurred both in superficial lining and in crypts of granulomatous colitis cases than in LPC. Lymphoplasmacytic colitis (group 3) still manifested histologic evidence of chronic inflammation, with a significant increased number of mononuclear cells in the mucosal chorion of bioptic samples (*p* < 0.001), while granulomatous colitis biopsies showed significantly increased neutrophils and macrophages (*p* < 0.001) interspersed in the lamina propria (Figure 2).

The Alcian PAS stain revealed an alteration of the chemical composition of mucin limited to the groups of adenocarcinoma and granulomatous colitis; a strong cytosolic PAS positivity was in fact observable, indicating a remarkable increase in the mucopolysaccharides amount if compared to controls or LPC samples (*p* < 0.001).

#### 3.1.1. Immunohistochemistry

Changes in polyamines cellular expression between groups were compared using Wilcoxon matched pairs tests. Resulting *p* values were corrected for multiple comparisons using the false discovery rate as described by Benjamini and Hochberg, and a *p* < 0.05 was considered significant. The positivity for the antigens ODC, PUT, and SPM had a cytosolic paranuclear localisation, while for the DAO antigen a basolateral positivity was appreciable according to what was observed by Oliva et al. [6]. No nuclear positivity was detectable for polyamines. The statistical elaboration of cellular counts for each polyamine in all the examined cases, compared to values obtained in control healthy dog, revealed a significant (*p* < 0.01) difference and a characteristic trend of the expression for every kind of lesion considered.

**Figure 1:**
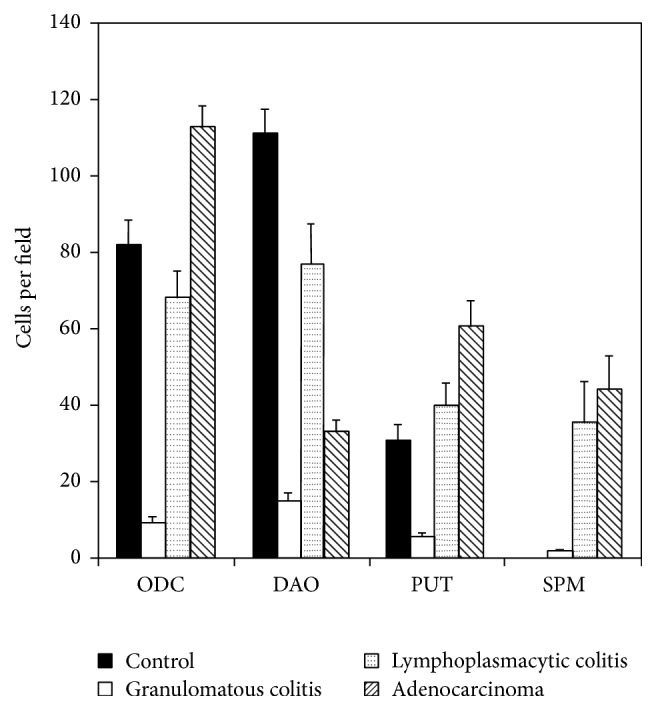


**Figure 2:**
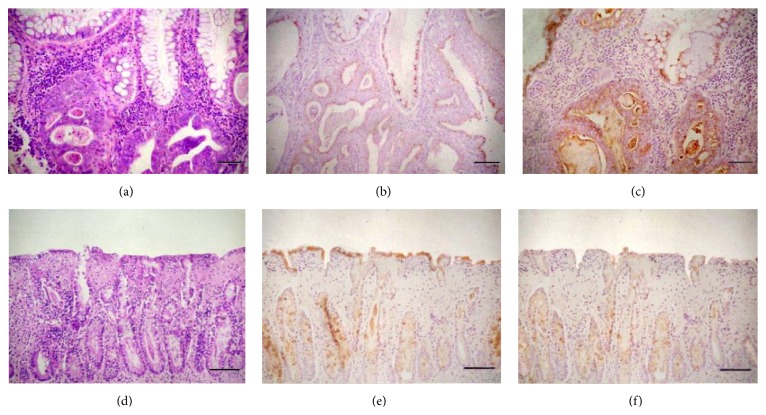
Expression of DAO and ODC in different colonic pathological conditions. (a) Morphological aspect of colonic carcinoma with areas of squamous metaplasia. (b) The same section stained with anti-DAO polyclonal antibody; note the weak positivity diffused particularly in well-preserved mucosal epithelium. (c) In a consequent section stained with anti-ODC pAb the strong expression of the enzyme is observed in metaplastic undifferentiated areas. (d) Morphology of granulomatous colitis with severe crypts involvement and areas of subepithelial fibrosis. (e) The expression of DAO in a consequent section shows a continuous and strong expression in the superficial epithelium, but only occasional and spotted strong stain in epithelium lining the crypts. (f) Weak and focal expression of ODC in a successive section evidences the low concentration of the enzyme in colonic epithelium during the granulomatous phlogosis. ((a) and (d) H&E; (b), (c), (e), and (f) IHC stain, Meyer's haematoxylin counterstain; (a), (b), and (c) bar = 300 *µ*m; (d), (e), and (f) bar = 600 *µ*m).

In both colitides (groups 2 and 3) the ODC antigen was “downexpressed” if compared to controls (*p* < 0.01; for both groups) (Figure 2(f)). In samples of adenocarcinoma the tendency was opposite (Figure 2(c)). The expression of PUT and SPM followed almost the same trend as ODC, both in neoplastic and in inflammatory lesions (data not shown). DAO antigen showed a significant lower expression (*p* < 0.001) in all kinds of lesions (Figures 2(b) and 2(e)), without specificity, than in controls as shown in Figure 1.

## 4. Discussion

The gastrointestinal (GI) tract is lined by a continuous layer ofepithelial cells which maintain the physical and functional barrier to undesirable luminal antigens [23]. Epithelial cells of mammalian GI mucosa rapidly proliferate and turn over approximately every three days under biological conditions [23, 24]. This process balances cell proliferation, differentiation, migration, and apoptosis. Polyamines (putrescine, spermidine, and spermine) have low molecular weight and are highly charged aliphatic polycations which are intimately involved in many distinct cellular functions. An increasing body of evidence has advanced our understanding of the cellular and molecular functions of polyamines especially in man and in mice/rats, used as principal animal models of GI inflammation and tumorigenesis. Until now, a substantial lack of information is observed regarding dogs. The goal of the present study was the evaluation, for the first time, of the presence and the pattern of expression of polyamines, their precursor and suppressor, in normal colonic conditions and in some pathological conditions frequently observed in this species. This preliminary study represents the first step to highlight the roles and mechanisms of cellular polyamines in dog GI mucosal pathology and also point out their potential clinical regulation in animals with mucosal injury-associated disorders.

Polyamine metabolism is involved with a wide variety of GI diseases. Numerous factors may influence polyamine homeostasis; however, the changes seem to be tissue-specific. The altered polyamine metabolism contributes to epithelial cell proliferation and esophageal carcinogenesis in experimental animals [25, 26]. In man, ODC activity is upregulated in Barrett's esophagus, a premalignant lesion, and correlated with the degree of dysplasia [27]. The relationship between polyamines and inflammatory bowel diseases (IBD) is extensively studied. Conflicting results are reported in different studies regarding the ODC activity and polyamine content in IBD patients. As observed in our data, in which decreased ODC levels are observed in both groups of dogs with different forms of colitis, it has been found that ODC activity was decreased in IBD patients in both involved and in uninvolved mucosal tissues [28]. This decrease was related to the severity of disease [28]. In contrast, ODC was found to be elevated both in human and in animal studies [29–31]. The discrepancy may be due to the content of tissue sample or, as in our cases of granulomatous colitis, due to the dramatic reduction/loss of epithelial cells in damaged areas, with a consequence of relative lowered levels of PAs expression [32, 33]. A few studies indicate that increased levels of spermidine and polyamine catabolism in IBD patients and in experimental models may relate to the accelerated proliferation of injured tissues [33, 34]. Spermine exerts an inhibitory role in the inflammatory reaction and is downregulated in severe ulcerative colitis patients and in chronic colitis experimental models [33, 35]. Our results are in agreement with these observations, demonstrating a significant and dramatic decrease of SPM in group 2, with respect to the levels of expression observed in group 3. It has been noticed that the decrease of spermine content may further aggravate the disease [33]. The role of polyamine metabolism in IBD is further supported by the fact that L-arginine improves colitis by enhancing the formation of polyamines in animal models while DFMO, an irreversible inhibitor of ODC, worsens the disease [33]. Pathological conditions outside the GI tract also closely relate to polyamine metabolism and interfere with internal polyamine pool. In colon cancer, the activities of polyamine-synthesizing enzymes and polyamine content are increased 3-4-fold compared to the equivalent normal colonic mucosa, and polyamines have even been attributed as markers of neoplastic proliferation in the colon [36]. However, the exact mechanisms on the role of internal polyamine pool affecting GI mucosal homeostasis are not yet clearly understood.

Our results suggest that, in the adenocarcinoma affected colonic dog mucosa, the balance between biosynthesis and degradation of polyamines may be disengaged. In fact, while ODC is strongly upregulated, DAO follows both an absolute and relative decrease, from a quantitative point of view, if compared, respectively, to control's levels of the same enzyme and to the levels of the other PAs.

On the other hand, the balance is conserved in inflammatory disease. Our main hypothesis about the trend of that balance in different class of diseases is based on the pathogenesis of the epithelial Noxa. In case of colitis both the upper and lower part of the cryptae are affected by the damage. The reduction in number of the mature enterocytes justifies the decrease of DAO which is an enzyme usually expressed in well-differentiated cells. The downexpression of ODC and PAs could be explained by the fact that the hyperplasia of the proliferative section does not supply from a quali-quantitative point of view the lack of cells, even if lymphoplasmacytic colitis PAs are higher than controls.

The neoplastic tissue is characterized by a population of immature cells, which produces a lower amount of DAO; at the same time, the exceptional rate of proliferation produces a large amount of newborn and highly immature cells that are able to express PAs. This evidence could explain the remarkable overexpression of ODC and PAs. Interestingly, almost all our markers, except SPM, have been found to be downregulated in IBD with respect to controls. Despite the fact that PAs and their acetylated derivatives are a prerequisite for cellular metabolism and considered to be essential for proliferation and differentiation of the rapidly renewing intestinal mucosa, their role during intestinal mucosal inflammation is less clear [33]. In some studies, a correlation of polyamine levels of patients with IBD with their corresponding inflammatory index revealed that increased concentrations of PAs were found in CECs from the most severe inflamed mucosal areas [33]. Using acute and chronic DSS colitis as a model of mucosal inflammation, the same authors found enhanced levels of PAs in acute forms, whereas in chronic inflammation, PAs concentrations were decreased [33]. Also our data indicate a lack of the anti-inflammatory PAs, especially spermine, in chronic colitis, which may aggravate the disease. In our opinion, the PAs biosynthetic pathway overexpression, reported in acute or chronic-active forms of intestinal inflammation, is one of the steps in the cancerogenic process; anyway, several investigations are requested.

The immunohistochemical evaluation of the polyamine's cycle in bioptic samples could be therefore useful in detecting the severity of some “borderline” lesions. A predictive value of these markers could be assessed by evaluating the group of dysplastic lesions, looking for a “cut-off” point in the quantitative expression between preneoplastic and nonneoplastic lesions.

The coherence of our results with the findings reported in human beings [37] could suggest a possible role of the dog with spontaneous disease as a model, especially in the evaluation of the efficacy of new therapeutic trials and protocols for adenocarcinoma.

## 5. Conclusion

Similar to humans, dogs express different intestinal levels of PAs, relative to different pathological conditions. We can hypothesize that contextual evaluation of ODC, DAO, and PAs could be suggestive of the severity of the lesion and in cases of overexpression a possible predictive factor of malignancy. While such a high polyamine supply may be of benefit in nonneoplastic colonic mucosal growth, the role of tissutal and luminal polyamines in colon cancer is a clear concern. The pool of PAs is taken up by neoplastic colonocytes, they are utilized in full to support neoplastic growth, and their uptake is strongly upregulated by the mitogens known to play an important role in colonic carcinogenesis. Inhibition of polyamine synthesis and their uptake, impaired utilization of exogenous polyamines, and enhanced catabolism of polyamines in neoplastic colonocytes may be therefore conceivable future approaches also in the chemoprevention of dog colorectal cancer.

## Figures and Tables

**Table 1:** Summary characteristics of enrolled dogs.

Group and race	Sex	Median age (range) in years
Group 1 (CTR) = 10 dogs		
Labradors (1)	m = 3, mn = 1, f = 2, fs = 4	6.3 (3–11)
Mix (4)		
Cocker (1)		
Beagle (2)		
German sheph. (2)		
Group 2 (GC) = 10 dogs		
Schnauzers (1)	m = 2, mn = 3, f = 1, fs = 4	6.4 (1–12)
Cocker (1)		
Yorkie (1)		
Mix (1)		
Rottweiler (1)		
Collie (1)		
Pekinese (1)		
Shih Tzu (1)		
Boxer (1)		
German sheph. (1)		
Group 3 (LPC) = 10 dogs		
Labradors (1)	m = 3, mn = 1, f = 1, fs = 5	4.9 (1–10)
Mix (2)		
Boxers (1)		
Rottweiler (1)		
Shih Tzu (1)		
Boxer (1)		
Siberian Husky (1)		
Basset-hound (1)		
German sheph. (1)		
Group 4 (Carc) = 10 dogs		
Shar pei (1)	m = 4, mn = 1, f = 2, fs = 3	9.9 (6–14)
Chow chow (2)		
Labrador (1)		
Boxer (1)		
Belgian sheph. (1)		
*Staffordshire bull terrier* (1)		
Mix (3)		

m* * = * *male, mn* * = * *neutered male, f* * = * *female, fs* * = * *spayed female; mix* * = not pure breed.

## References

[1] Pegg A. E. (2009). Mammalian polyamine metabolism and function. *IUBMB Life*.

[2] Rial N. S., Meyskens F. L., Gerner E. W. (2009). Polyamines as mediators of APC-dependent intestinal carcinogenesis and cancer chemoprevention. *Essays in Biochemistry*.

[3] Fujiwara K., Masuyama Y., Kitagawa T. (1996). Immunocytochemical localization of polyamines in the gastrointestinal tracts of rats and mice. *Histochemistry and Cell Biology*.

[4] Linsalata M., Russo F., Cavallini A., Berloco P., Di Leo A. (1993). Polyamines, diamine oxidase, and ornithine decarboxylase activity in colorectal cancer and in normal surrounding mucosa. *Diseases of the Colon & Rectum*.

[5] Matsubara N., Hietala O. A., Gilmour S. K. (1995). Association between high levels of ornithine decarboxylase activity and favorable prognosis in human colorectal carcinoma. *Clinical Cancer Research*.

[6] Oliva F., Cortese L., Papparella S., D'Agostino L., Daniele B., Perschino A. (1997). Postheparin plasma diamine oxidase and its intestinal location in dog. *The European Journal of Comparative Gastroenterology*.

[7] Raithel M., Ulrich P., Hochberger J., Hahn E. G. (1998). Measurement of gut diamine oxidase activity: diamine oxidase as a new biologic marker of colorectal proliferation?. *Annals of the New York Academy of Sciences*.

[8] Gerner E. W., Meyskens F. L. (2004). Polyamines and cancer: old molecules, new understanding. *Nature Reviews Cancer*.

[9] Zaletok S., Alexandrova N., Berdynskykh N. (2004). Role of polyamines in the function of nuclear transcription factor NF-kappaB in breast cancer cells. *Experimental Oncology*.

[10] Simpson K. W., Jergens A. E. (2011). Pitfalls and progress in the diagnosis and management of canine inflammatory bowel disease. *Veterinary Clinics of North America—Small Animal Practice*.

[11] Cerquetella M., Spaterna A., Laus F. (2010). Inflammatory bowel disease in the dog: differences and similarities with humans. *World Journal of Gastroenterology*.

[12] Jergens A. E., Schreiner C. A., Frank D. E. (2003). A scoring index for disease activity in canine inflammatory bowel disease. *Journal of Veterinary Internal Medicine*.

[13] German A. J., Helps C. R., Hall E. J., Day M. J. (2000). Cytokine mRNA expression in mucosal biopsies from German shepherd dogs with small intestinal enteropathies. *Digestive Diseases and Sciences*.

[14] Day M. J., Bilzer T., Mansell J. (2008). International standards for the histopathological diagnosis of gastrointestinal inflammation in the dog and cat: a report from the World Small Animal Veterinary Association Gastrointestinal Standardization Group. *Journal of Comparative Pathology*.

[15] Cohen S. S. (1997). *A Guide to the Polyamines*.

[16] Kurien B. T., Dorri Y., Dillon S., Dsouza A., Scofield R. H. (2011). An overview of western blotting for determining antibody specificities for immunohistochemistry. *Methods in Molecular Biology*.

[17] Jergens A. E. (1999). Inflammatory bowel disease: current perspectives. *Veterinary Clinics of North America—Small Animal Practice*.

[18] Allenspach K., Wieland B., Gröne A., Gaschen F. (2007). Chronic enteropathies in dogs: evaluation of risk factors for negative outcome. *Journal of Veterinary Internal Medicine*.

[19] Dixon M. F., Genta R. M., Yardley J. H. (1996). Classification and grading of gastritis: the updated Sydney system. *The American Journal of Surgical Pathology*.

[20] Head K. W., Else R. W., Dubielzig R. R., Meuten D. J. (2002). Tumors of the alimentary tract. *Tumors in Domestic Animals*.

[21] Head K. W., Cullen J. M., Dubielzig R. R. (2003). *Histological Classification of Tumors of the Alimentary System of Domestic Animals*.

[22] Poletti A., Giacon C., Pennelli N. (1992). Simultaneous visualization of immunodetected antigens and tissue components revealed by non-enzymatic histochemical stains. *Journal of Histochemistry and Cytochemistry*.

[23] Catalioto R.-M., Maggi C. A., Giuliani S. (2011). Intestinal epithelial barrier dysfunction in disease and possible therapeutical interventions. *Current Medicinal Chemistry*.

[24] Timmons J., Chang E. T., Wang J.-Y., Rao J. N. (2012). Polyamines and gut mucosal homeostasis. *Journal of Gastrointestinal & Digestive System*.

[25] Pera M., Grande L., Gelabert M. (1998). Epithelial cell hyperproliferation after biliopancreatic reflux into the esophagus of rats. *Annals of Thoracic Surgery*.

[26] Gerner E. W., Garewal H. S., Emerson S. S., Sampliner R. E. (1994). Gastrointestinal tissue polyamine contents of patients with Barrett's esophagus treated with alphadifluoromethylornithine. *Cancer Epidemiology Biomarkers and Prevention*.

[27] Garewal H. S., Sampliner R. (1989). Barrett's esophagus: a model premalignant lesion for adenocarcinoma. *Preventive Medicine*.

[28] Ricci G., Stabellini G., Bersani G. (1999). Ornithine decarboxylase in colonic mucosa from patients with moderate or severe Crohn's disease and ulcerative colitis. *European Journal of Gastroenterology and Hepatology*.

[29] Pillai R. B., Tolia V., Rabah R., Simpson P. M., Vijesurier R., Lin C.-H. (1999). Increased colonic ornithine decarboxylase activity in inflammatory bowel disease in children. *Digestive Diseases and Sciences*.

[30] Pegg A. E., Wechter R., Pakala R., Bergeron R. J. (1989). Effect of N1,N12-bis(ethyl)spermine and related compounds on growth and polyamine acetylation, content, and excretion in human colon tumor cells. *The Journal of Biological Chemistry*.

[31] Gobert A. P., Cheng Y., Akhtar M. (2004). Protective role of arginase in a mouse model of colitis. *Journal of Immunology*.

[32] Marcuard S. P., Silverman J. F., Finley J. L., Seidel E. R. (1992). Ornithine decarboxylase activity during gastric ulcer healing in dogs. *Digestive Diseases and Sciences*.

[33] Weiss T. S., Herfarth H., Obermeier F. (2004). Intracellular polyamine levels of intestinal epithelial cells in inflammatory bowel disease. *Inflammatory Bowel Diseases*.

[34] Obayashi M., Matsui-Yuasa I., Matsumoto T., Kitano A., Kobayashi K., Otani S. (1992). Polyamine metabolism in colonic mucosa from patients with ulcerative colitis. *American Journal of Gastroenterology*.

[35] Mössner J., Hammermann R., Racké K. (2001). Concomitant down-regulation of L-arginine transport and nitric oxide (NO) synthesis in rat alveolar macrophages by the polyamine spermine. *Pulmonary Pharmacology & Therapeutics*.

[36] Milovic V., Turchanowa L. (2003). Polyamines and colon cancer. *Biochemical Society Transactions*.

[37] Seiler N., Atanassov C. L., Raul F. (1998). Polyamine metabolism as target for cancer chemoprevention. *International Journal of Oncology*.

